# Supplementary material for: The river Rhine transports around 4,000 tonnes of macrolitter towards the North Sea each year
Source: Commun Sustain. 2026 Jan 8;1(1):5. doi: 10.1038/s44458-025-00007-5 (PMC12863643; doi:10.1038/s44458-025-00007-5)
Supplement: Supplementary file 2 — Supplementary Information [file 44458_2025_7_MOESM2_ESM.pdf]

The river Rhine transports around 4,000 tons of macrolitter towards the North Sea each year

SUPPLEMENTARY INFORMATION

Nina Gnann[1+], Katharina Höreth[2+], Nicolas Schweigert[3], Mariele Evers[2], Thomas A. Ternes[4], Leandra Hamann[5*]

1 Department of Geosciences, Eberhard Karls University Tübingen, 72070 Tübingen, Germany
2 Department of Geography, University of Bonn, 53115 Bonn, Germany
3 K.R.A.K.E. (Kölner Rhein-Aufräum-Kommando-Einheit) e.V., 51105 Cologne, Germany
4 Federal Institute of Hydrology, Am Mainzer Tor 1, Koblenz 56068, Germany
5 Bonn Institute for Organismic Biology, Section 2, Animal Diversity, University of Bonn, 53121 Bonn, Germany

* Corresponding author: lhamann@biob.uni-bonn.de
[+] These authors contributed equally

Supplementary Notes 1

Environmental parameters: Measurements and results

The litter trap (LT) is located at the west side of the Rhine, along the concave slope where the river bank is heavily reinforced. The steep river bank on that side is secured with large stones, which contrast with the water surface on satellite imagery, that aids the identification of the water line (Figure SI-1). When the water level exceeds the bank full discharge at this specific location, a tarred shore area of 20 m width becomes flooded until the water reaches the next reinforced wall. The convex slope on the opposite river side to the east rises gently over a gravel bed and transitions into a public green area.

To determine the Rhine's width at the LT location under varying water levels, satellite imagery of different dates was downloaded from planet.com. A total of 21 satellite images from 2018 to 2023, corresponding to mean daily discharges between 868 m^3 and 5030 m^3, were visually analysed. Each satellite image was assigned a minimum and maximum river surface width, due to uncertainties caused by trees, object shadows, or wetted surface ground that hindered the identification of the water line and the image resolution of 3 m. This led to a deviation of the river width in single satellite images of 11.8 m (mean). Based on the 21 selected satellite images, the river width varied between a minimum of 296.7 m and a maximum of 400.6 m.

To determine the Rhine's width at the mean water level of 283.1 cm during the statistical year, five of the 21 selected satellite images were chosen from days when the water level was within +/- 5 cm of this value. Therefore, the mean river width of the five images was determined as 326.7 ± 13.8 m (Figure SI-1).

The flow velocity was measured at three points, i.e. at the entrance, in the upstream basket, and in the downstream basket of the LT (Figure SI-1B). Measurements were taken using a water flow metre (OTT MF pro, 0-6 m s^{-1}, ±2 % accuracy) at depths ranging from 0.1 to 0.9 m, before and after cleaning, across six sampling dates in 2024. Mean and standard deviation were calculated for each position before and after cleaning averaging across all depths. At the LT entrance, the flow velocity was 0.67 ± 0.1 m s^{-1} before cleaning and 0.70 ± 0.2 m s^{-1} after cleaning (N = 5 sampling dates, one date was excluded because of a tree at the entrance). This equals an average velocity of 0.66 ± 0.15 m s^{-1} at the entrance. In the upstream basket, the flow velocity was 0.75 ± 0.12 m s^{-1} before cleaning and 0.64 ± 0.4 m s^{-1} after cleaning (N = 6 sampling dates). In the downstream basket, flow velocity was 0.09 ± 0.19 m s^{-1} before and 0.47 ± 0.17 m s^{-1} after the cleaning of the downstream basket (N = 6 sampling dates) (Figure SI-1B).

Supplementary Methods 1

Description of final dataset, monitoring and validation process

The results of the on-site monitoring (Supplementary Figure 2G) are directly uploaded into an online spreadsheet by using the ODK-App. Each row of this spreadsheet contains one or several items of one category. Counted objects comprise all tangible ML items within the LT baskets larger than one centimetre. The measurements carried out between September 24, 2022 and November 19, 2022 were considered a learning phase. Each row of the dataset was validated by two authors (NG and KH) to minimise errors, increase uniformity between monitoring volunteers, and categorise on-site unknown objects. Difficult cases were discussed and validated by three authors (NG, KH, and LH). Supplementary Table 1 shows the rules of validation set by the authors. If a change to the original dataset was done, the change was marked in an extra column called "validation notes" of the final dataset (see supplement raw dataset 20250111_Data.xlsx, sheet name: LT ML Data, link: https://doi.org/10.5281/zenodo.17108281).

During the validation process, we encountered the difficulties that were solved as followed:

a. The material, usage type and (sub-)category are the core of the monitoring and within the validation process. Due to items being made of mixed materials, the largest material component of an object justifies its categorization. For example: Wooden rocket sticks count as wooden fireworks despite the small plastic remains of the rocket.
b. ML categories with high inter-class (two or more optically similar categories, difficult to discriminate) or high intra-class variance (one category, which contains very different items). Examples are:
    i. high inter-class variability: e.g. shoes and sandals made of leather and/or textile, where a possible overlap with footwear made of plastic - not flip flops (mixed fibres/ artificial leather/ shoe soles) exist.
    ii. high intra-class variability: pl_re_toy_ covers plastic balls, sandpit buckets, glitter stickers, etc.
c. During the monitoring process, ML of the same category should have been separated by size and recorded separately. However, this was not consequently done due to capacity reasons. Therefore, the column "size definition" was added during validation, meaning:
    i. unambiguous, if definition a, b, c, … applies.
    ii. mixed, if definition as < a, < b, < c, … applies
    iii. unknown, if size classes were not validated.

d. The weight measured for each item on site could not be validated afterwards. Therefore, the column “Content” depicts if the measured weight is valid (0), invalid (1) or missing (2). Large wooden items could not be transported from the sampling site to the monitoring site. Therefore, the estimated weight is considered as invalid. Some items were filled or soaked with liquids. Those items' weights also count as invalid. Some items weigh less than the scale could measure (1 g). In most cases items were weighted together and then the weight divided by the number to derive their weight below 1 g.

Supplementary Figure 1

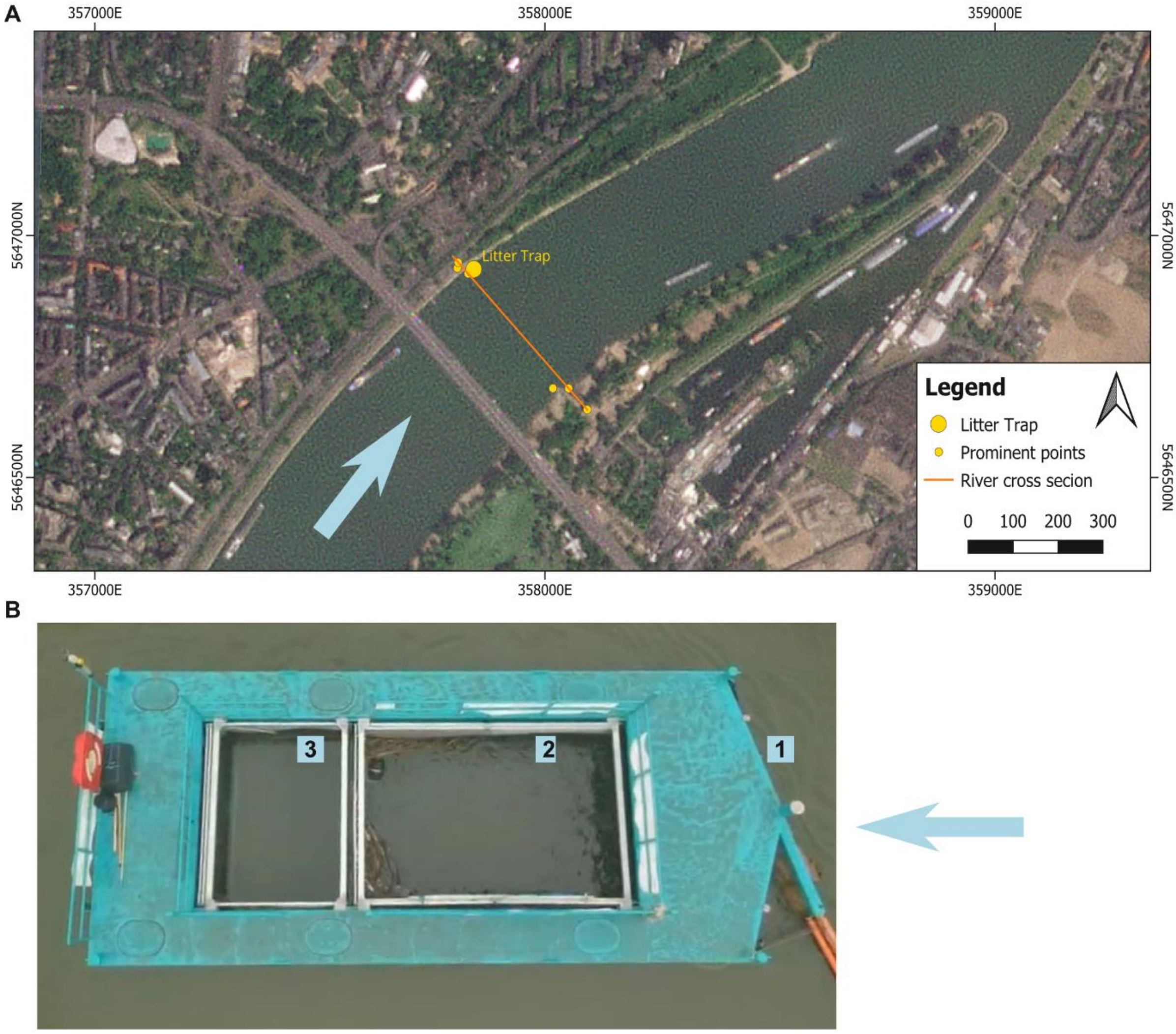

Supplementary Figure 1: LT position and measurement of environmental parameters. A) River cross section at LT visible on a satellite picture (planet.com) on 30.05.2023 with a water level of 280 cm and a discharge of 1760 $m^3 s^{-1}$. B) Measurement points for flow velocity marked in a top view of the cleaned LT.

Supplementary Figure 2

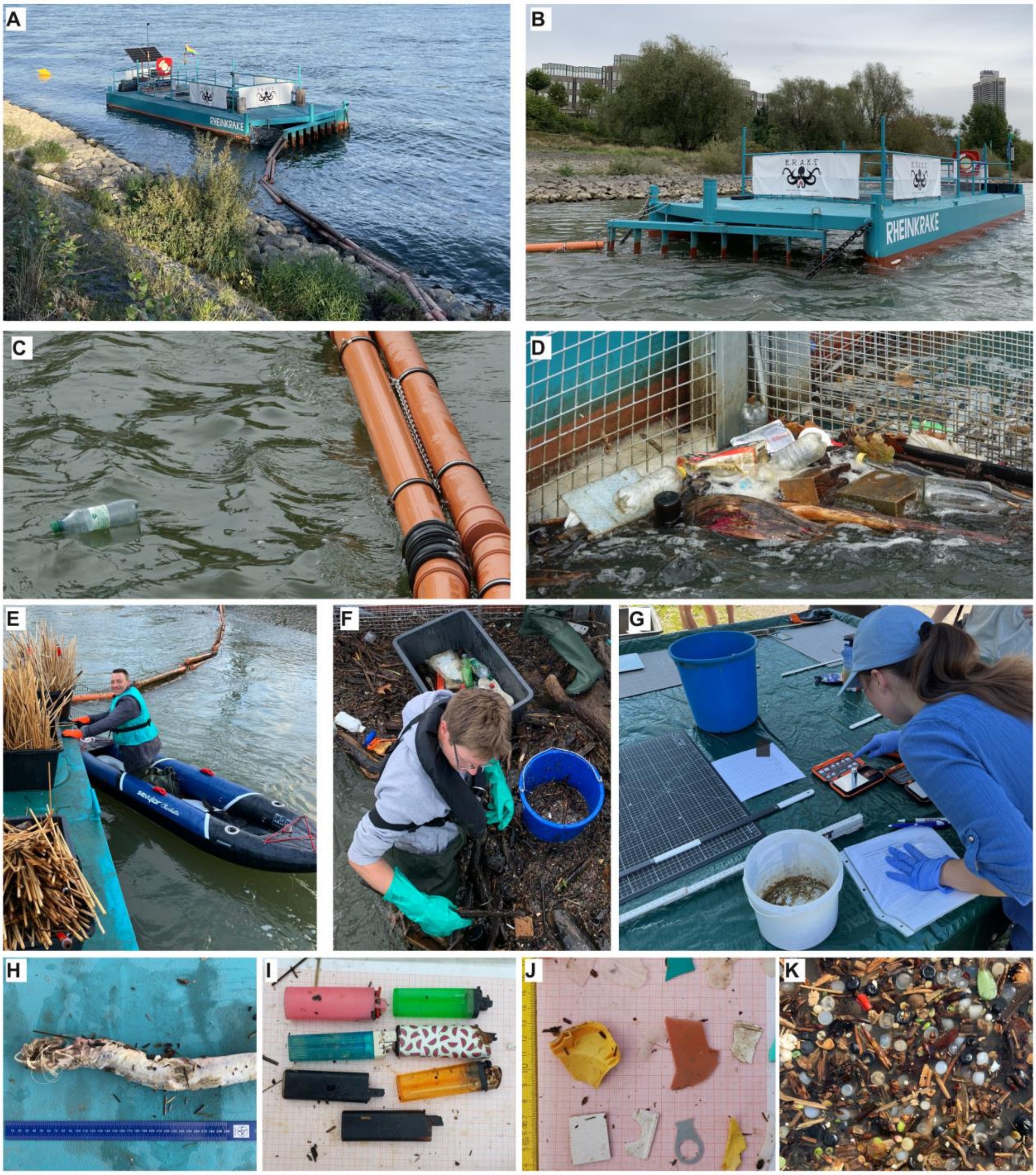

Supplementary Figure 2: A) LT with the floating boom that connects to shore. B) Entrance of the LT with protecting rakes that retain large branches and trees. C) The floating boom guides ML towards the entrance of the LT. D) The LT is very full and some ML is already in the collecting baskets. E) The LT is approached by boat or kayak every two weeks for cleaning and sampling. F) Volunteers wear wading pants to retrieve ML from the basket and collect in plastic boxes. Dead wood and non-anthropogenic debris was removed during cleaning and not transported to the monitoring site. G) After the ML was transported to shore, volunteers sort and categorise each item using the customised app. H) Dead and alive animals are also

measured and monitored. I) Lighters are a common ML item that is easy to identify. J) Non-foamed plastic fragments are the third most common category found in the river Rhine. K) Even though items <3.818 cm are not systematically retained by the litter trap, small litter, such as plastic pellets can often be found in the LT.

Supplementary Table 1

Supplementary Table 1: Overview of validation rules used in this study.

Comment	Action
Corrected (count)	Countable number has been corrected based on information of image (or weight)
Invalid/ corrected (count)	Uncountable number, Number was reduced to visible plausible count in case original count is > 150% of validated count. Those typos happened due to weight and number fields being too close to each other.
Plausible (count)	Items not countable on image, but plausible number (mismatch < 150%)
Recategorised (category)	Category has been corrected.<br><br>The knowledge of the citizens experienced in clean-ups was also valuable in the validation process and their knowledge was used by sharing pictures of unknown objects in the shared Whatsapp group.
No image, plausible	Category, Weight, and Count were checked and are plausible
Contains items < 2.5 cm	Items of size 1 – 2.5 cm were counted and marked
Contains items > 1 m	Items of size > 1 m were counted and marked

Supplementary Table 2

Supplementary Table 2: Spearman Rank Correlation Coefficients between the environmental factors and A) eight material categories by count (organic food waste is missing due to insufficient significance level) and B) categories by count that show a correlation coefficient ρ > 0.7 and C) size classes defined as unambiguous according to size definition. See supplement statistics code script for category-wise ρ values. Reported values are statistically significant results ($p < 0.05$).

		rising discharge in $m^3\ s^{-1}$	discharge in $m^3\ s^{-1}$	wind speed in $m\ s^{-1}$	precipitatio n in mm	temperature in °C
		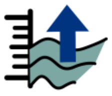	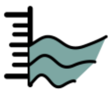	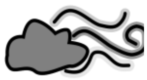	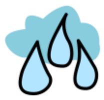	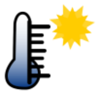
**A**	plastic	**0.78**	**0.75**	0.50	0.43	-
	worked wood	**0.89**	**0.70**	0.54	0.67	-
	glass & ceramics	0.64	-	-	0.55	-
	paper & cardboard	0.51	0.42	-	0.42	-
	metall	**0.78**	0.64	0.47	0.55	-
	rubber	**0.78**	0.66	0.61	0.54	-

	chemicals	0.60	-	0.40	0.56	-
	cotton & textiles	0.61	-	-	0.65	-
**B**	glass light bulbs	**0.81**	0.65	0.52	0.48	-
	metal foil wrappers	**0.78**	0.67	0.51	0.57	-
	plastic drink bottles ≤ 0.5 l	**0.72**	0.43	0.50	0.65	-
	plastic caps/lids drinks	**0.78**	0.64	0.56	0.47	-0.43
	plastic lolly & ice-cream sticks	**0.87**	0.66	0.53	0.48	-0.43
	plastic single-use face-mask	0.55	0.62	0.51	-	**-0.76**
	fragments of foamed polystyrene 2.5 cm ≥ ≤ 50 cm	**0.80**	**0.77**	0.47	0.4	-
	fragments of non-foamed plastic 2.5cm ≥ ≤ 50cm	**0.77**	**0.75**	0.42	0.4	-
	other identifiable foamed plastic items	0.64	**0.71**	-	-	-
	plastic cigarette lighters	**0.83**	**0.74**	0.56	0.46	-
	rubber condoms (incl. packaging)	**0.72**	0.4	0.56	0.61	-
	other rubber pieces	**0.73**	0.59	0.51	0.54	-
	wooden corks	**0.88**	0.64	0.51	0.65	-0.39
	other processed wooden items 2.5 cm ≥ ≤ 50 cm	**0.84**	0.55	0.45	**0.70**	-
	wooden fireworks & matches	0.51	**0.74**	0.41	-	-0.43
**C**	samples of size (1)/2.5 - 5 cm*	**0.83**	0.66	0.45	0.57	-
	samples of size 5 - 10 cm*	**0.82**	**0.75**	0.48	0.47	-
	samples of size 10 - 20 cm*	**0.73**	**0.73**	0.55	0.46	-
	samples of size 20 - 30 cm*	0.65	0.48	-	0.44	-
	samples of size 30 - 50 cm*	0.55	-	0.42	-	-
	samples of size >50 cm*	0.55	0.5	-	-	-

* samples with unambiguous classification in column “Size_Definition” only
